# HPLC-DAD phenolic screening and *in vitro* assessment of antimicrobial, antioxidant and anti-inflammatory activities of Tanteboucht dates

**DOI:** 10.1039/d2ra01630c

**Published:** 2022-05-04

**Authors:** Saliha Dassamiour, Selsabil Meguellati, Hdouda Lamraoui, Mohamed Sabri Bensaad, Rokayya Sami, Garsa Alshehry, Eman Hillal Althubaiti, Areej Suliman Al-Meshal

**Affiliations:** Laboratory of Biotechnology of Bioactive Molecules and Cellular Physiopathology (LBMBPC), Department of Microbiology and Biochemistry, Faculty of Natural and Life Sciences, University Batna 2 Fesdis Batna 05078 Algeria s.dassamiour@univ-batna2.dz m.bensaad@univ-batna2.dz; Department of Microbiology and Biochemistry, Faculty of Natural and Life Sciences, University Batna 2 Fesdis Batna 05078 Algeria katrykorina@gmail.com onzukaeikich87@yahoo.com; Laboratory of Cellular and Molecular Physio-Toxicology-Pathology and Biomolecules (LPTPCMB), Faculty of Natural and Life Sciences, University Batna 2 Fesdis Batna 05078 Algeria; Department of Food Science and Nutrition, College of Sciences, Taif University P.O. 11099 Taif 21944 Saudi Arabia rokayya.d@tu.edu.sa garsa.a@tu.edu.sa; Department of Biotechnology, Faculty of Science, Taif University P.O. 11099 Taif 21944 Saudi Arabia i.althubaiti@tu.edu.sa; Department of Biology, College of Science and Humanities in Al-Kharj, Prince Sattam bin Abdulaziz University Al-Kharj 11942 Saudi Arabia a.almashal@psau.edu.sa

## Abstract

The date palm (*Phoenix dactylifera* L.) is one of the most important crops in arid and semi-arid zones. Date fruit occupies a good place in traditional medicine among the Saharan residents, due to its therapeutic virtues; although there may be several therapeutic virtues yet to be discovered. The aim of this study was to investigate the phytochemical and pharmacological properties of the hexanic (EHx), chloroformic (ECh), ethyl acetate (EAc) and aqueous (EAq) extracts of Tanteboucht pulp. The phytochemical characterization and estimation of phenolic compounds were done based on an HPLC-DAD approach. The antioxidant activity was evaluated by a DPPH scavenging effect test. The sensitivity of 7 bacterial strains and *Candida albicans* to Tanteboucht extracts was tested using the diffusion disc on agar medium method. The membrane stabilization test was used to determine the *in vitro* anti-inflammatory effect of the fruit extracts. Fourteen phenolic compounds were detected in organic extracts and EAc was the richest followed by ECh and finally EHx which was very poor in these molecules. All extracts showed antioxidant, anti-inflammatory and antimicrobial properties which differ in rate. Indeed, ECh had the greatest scavenging effect on DPPH, followed by EAc and then EAq. EAc was the most potent inhibitor of microbial strains. EAc and ECh were more efficient at membrane stabilization followed by EAq and the three extracts had more anti-inflammatory capacity than the positive control acetyl salicylic acid. The obtained considerable activities were significantly correlated with flavonoid and tannin contents in the extracts.

## Abstract 2

The date palm (*Phoenix dactylifera* L.) is one of the most important crops in arid and semi-arid zones.

## Graphical abstract

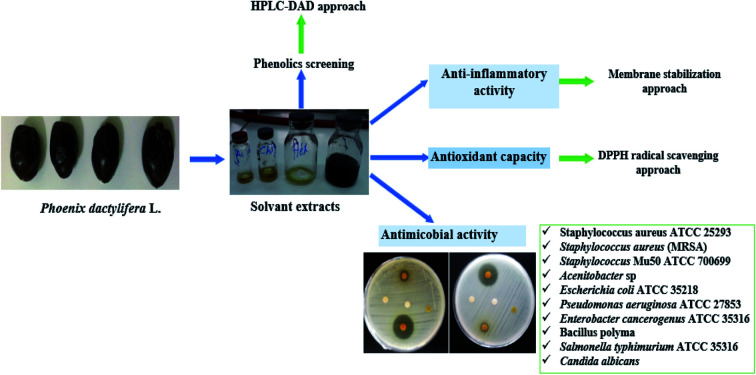

## 1. Introduction

At the present time the majority of the inhabitants of the terrestrial globe use a lot of plants as remedies in traditional medicines. Crude plant extracts are gaining interest as a potential source of bioactive natural molecules. They are being used for the treatment of infectious diseases and the protection of food against oxidation.^1^

The date palm (*Phoenix dactylifera* L.) is one of the most important crops in arid and semi-arid zones, and plays an important role in the economic and social life of the populations of these regions.^2^ It is one of the fruit species whose cultivation has existed since ancient times.^3^

The date is a fruit obtained from the date palm, has significant pharmaceutical, nutritional and commercial value. In addition to its high energy value^4^ due to its high carbohydrate content, date pulp is rich in phytochemicals such as phenolic compounds, especially anthocyanins, procyanidins, flavonoids and phenolic acids. These compounds are the subject of increasing interest^5,6^ besides their pharmacological properties. Indeed, several activities such as antioxidant, antimicrobial, anti-inflammatory and anti-tumour activities are attributed to these metabolites.^7–9^ The latter remain less defined in the case of dates in general and that of its common varieties in particular. Tanteboucht, which is one of these varieties, is the subject of this study where we (the authors) targeted its phytochemical composition and its related biological activities.

## 2. Experimental

### 2.1. Plant material

**Fig. 1:**
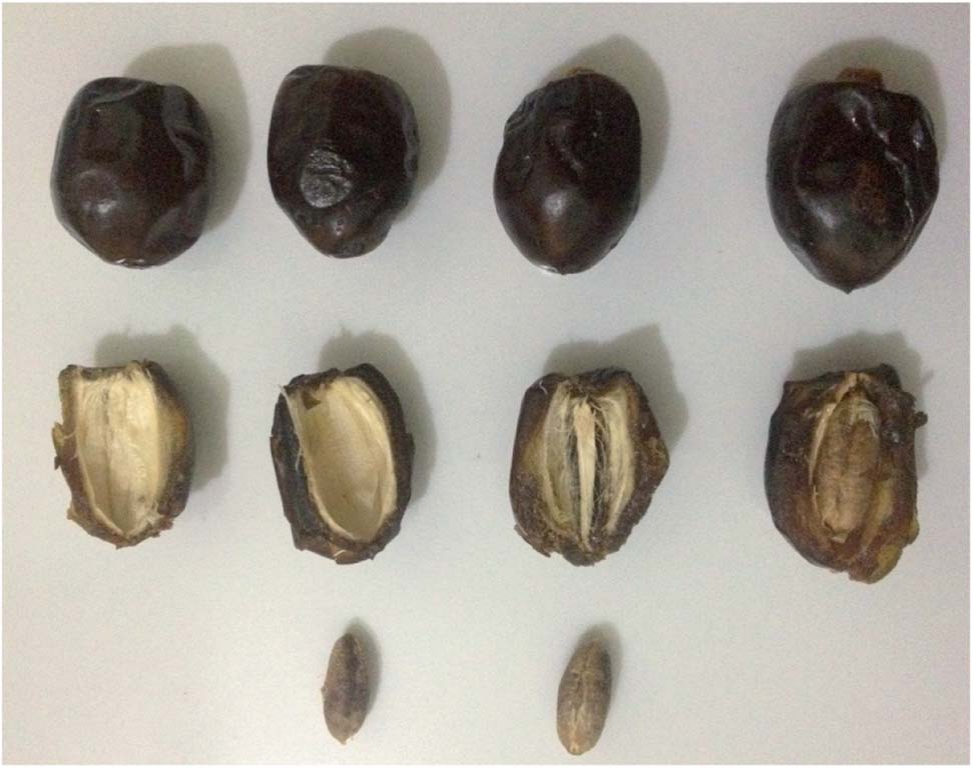
Tanteboucht date.

The used variety of date (*Tanteboucht*) is a semi-soft fruit one, growing in the south-eastern region (Ziban) of Algeria (Fig. 1). The fruits were harvested in October (Which year???); samples were sorted and stored in the refrigerator until the time of physiochemical analysis, in order to slow down chemical and physiological changes.

### 2.2. Extracts preparation

The crude extract was prepared by macerating 1500 g of pitted and crushed samples into 4 L of the mixture of acetone/water (60/40). The mixture was stored in a dark environment for 24 h before it was filtered. The obtained filtrate was then concentrated by evaporation of acetone at 37 °C. The remaining aqueous phase was used for carrying out a liquid–liquid extraction with successively three organic solvents of increasing polarity, hexane, chloroform and ethyl acetate to ensure good fractionation of the molecules. Dry extracts, obtained after evaporation of the three solvents respectively, EHx, ECh and EAc were used, in addition to the raw aqueous extract (EAq), to carry out the assays and the tests of the biological activities.

### 2.3. Microbial strains

The bacterial strains used in this study were: of Gram-positive bacteria: *Staphylococcus aureus* ATCC 25293, methicillin-resistant *staphylococcus aureus* (MRSA), *Staphylococcus Mu50* ATCC 700699; and Gram-negative bacteria: *Acenitobacter* sp, *Escherichia coli* ATCC 35218, *Pseudomonas aeruginosa* ATCC 27853, *Enterobacter cancerogenus* ATCC 35316, *Bacillus polyma, Salmonella typhimurium* ATCC 35316 besides a fungal strain ‘*Candida albicans*’.

The microbial strains were obtained from the microbiology laboratory of the University of Batna 1-Algeria.

### 2.4. HPLC-DAD phenolics screening

HPLC analysis was carried out by an AGILENT TECHNOLOGY apparatus. The extracts (EHx, ECh and EAc) used in this assay were dissolved in extraction solvents and injected in a volume of 15 μL each, onto an RP-C18 column set at a temperature of 35 °C. The mobile phase consisted of a mixture of acetonitrile (ACN)/methanol/acidified water. Elution was carried out in a gradient at a flow rate of 0.5 mL min; spread over 70 min of time. The gradient program was started with 5% of ACN and 95% acidified H_2_O for the first 5 min and then increased gradually to 60% till 30 min for ACN before bringing it down again to 5%, but decreased gradually to 15% for acidified H_2_O. Whereas the methanol starting addition was of 10% at 30 min and increased to 80% at the end of analysis.

Detection was performed at three different wavelengths, 250 nm, 280 nm and 340 nm.^10^

### 2.5. Tests of the biological activities of the extracts

#### 2.5.1. Evaluation of antioxidant activity by DPPH radical scavenging effect

The DPPH radical scavenging effect was evaluated as described by Mansouri *et al.*^11^ The concentrations of the extracts in the reaction medium were from 0.005 to 10 mg mL; while for standard antioxidants (Quercetin, vitamin C), they were from 0 to 50 μg mL^−1^. Extracts and standards were dissolved in methanol and a volume of 25 μL of each solution was added to 975 μL DPPH solution, the mixture was left in the dark for 30 min and the discoloration compared to negative control containing only the DPPH solution was measured at 517 nm. The following equation was used to calculate the DPPH scavenging activity:
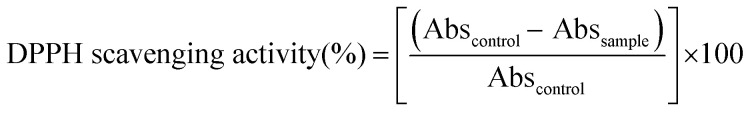
where, Abs_control:_ is the absorbance of control group, and Abs_sample_: is the absorbance of plant sample.

In addition, the anti-free radical efficacy is calculated as follows: EA = 1/IC_50_

Where the IC_50_ is the concentration of extract required to obtain 50% of the reduced form of the DPPH radical.

#### 2.5.2. Study of antimicrobial activity

Antimicrobial screening was performed using five concentrations for ethyl acetate extract; (50%, 25%, 12.5%, 6.25%, 3.12%); and a one concentration for the others. Organic extracts were dissolved in DMSO, while aqueous extract was dissolved in sterile distilled water. The test was carried out as described by Daas amiour *et al.*^12^ After preparation of the inoculum and swabbing, Whatman paper discs, of 6 mm diameter, were impregnated with 10 μl of the extract and placed on inoculated Mueller Hinton agar for bacteria and sabouraud for the fungal strain. Cultures were incubated at 37 °C for 24 h for bacterial species and 48 h for fungal species. The antibiograms were read using a calliper. An extract is considered active when a zone of inhibition is measured around the disc with a diameter greater than 6 mm and inside which no bacterial or fungal growth is observed.

#### 2.5.3. *In vitro* membrane stabilizing effect

*In vitro* membrane stabilization activity was performed using the hypotonicity method inducing lysis of the human erythrocyte membrane described by Shinde *et al.*^13^ The used blood was obtained from the CHU blood transfusion center of Batna; it was collected on tubes containing EDTA anti-coagulant from healthy donors. In this test, the whole blood was centrifuged at 3000 rpm for 10 min. The pellet was collected and rinsed with a mixture of a buffered isotonic solution (0.9 M NaCl in 0.15 M of phosphate buffer (pH 7.4)). The operation was repeated three times and each time the erythrocyte solution was centrifuged at 3000 rpm for 10 min. The test mixture contains 1 mL of phosphate buffer, 2 mL of hypo-saline solution (0.2%), 0.5 mL of the erythrocyte suspension [10%, v/v] with 0.5 mL of extract dissolved in physiological water (2 mg NaCl mL^−1^). For positive and negative controls, the extract was replaced by acetyl salicylic acid and distilled water respectively. After incubation at 56 °C for 30 min and centrifugation, the hemoglobin content in the supernatant was estimated by spectrophotometer at 540 nm. The percentage of hemolysis in the presence of distilled water is 100%. The stabilization percentage of the erythrocyte membrane is calculated using the formula:
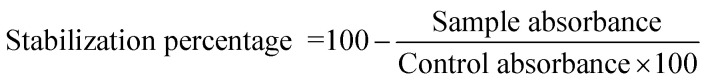


### 2.6. Statistical analysis

The experiments were carried out in triplicate and Results were expressed as mean ± standard deviation (*n* = 3) for each case. Statistical analysis was performed using one-way analysis of variance (ANOVA) followed by multiple Tukey's comparison tests. Differences were considered significant at *P* <0.001.

## 3. Results and discussion

### 3.1. Results of HPLC analysis

Identified phenolic acids in Tanteboucht extracts were gallic, caffeic, chlorogenic and 4-hydroxybenzoic acids. The presence of these six flavonoids was highlighted in the ECh and EAc extracts as: rutin, quercetin, luteolin, catechin, epicatechin and cyanidine chloride, besides the coumarin and vanillin compounds. Furthermore, tannic acid and procyanidin B2 were detected as hydrolysable and condensed tannins respectively (Table 1).

**Table 1:** Contents of detected phenolic compounds^a^

Phenolic compounds	Content (μg g^−1^) of EHx	Content (μg g^−1^) of ECh	Content (μg g^−1^) of EAc	En μg/100 g of FFw
Gallic acid	—	11.27 ± 0.35	8.91 ± 1.33	60.38 ± 5.72
Caffeic acid	—	12.89 ± 0.16	5.94 ± 0.79	53.58 ± 3.27
Chlorogenic acid	—	40.28 ± 2.79	32.47 ± 3.75	218.08 ± 20.55
4-Hydroxybenzoic acid	—	26.87 ± 1.77	4051.10 ± 71.98	14 812.63 ± 266.4
Coumaric acid	—	—	—	
*Trans*-ferulic acid	—		—	
Salicylic acid	—	—	—	
Vanillin	1.83 ± 0.17	29.68 ± 1.39	—	95.16 ± 3.60
Coumarin	50.59 ± 5.74	22.58 ± 2.02	—	107.64 ± 10.42
Rutin	—	21.02 ± 1.60	—	52.13 ± 3.96
Quercetin	—	—	207.05 ± 25.55	753.66 ± 93.02
Catechin	—	19.69 ± 2.91	17.01 ± 2.62	110.75 ± 16.75
Epicatechin	—	14.65 ± 2.02	—	36.33 ± 5.02
Luteolin	—	—	12.69 ± 1.85	46.19 ± 6.74
Cyanidine chloride	—	20.02 ± 4.48	4.26 ± 1.75	65.16 ± 17.50
Tannic acid	—	7.25 ± 2.15	8.11 ± 1.61	47.5 ± 11.20
Procyanidine B2	—	6.85 ± 0.78	4.20 ± 0.49	32.28 ± 3.73

a ‘—’: absent; FFW: fruit fresh weight.

These results are in agreement with those earlier found by Mansouri *et al.*^11^ for the same date variety; where they highlighted the presence of flavonoids and phenolic acids as caffeic acid and derivatives of cinnamic acid. It has been observed that Gallic acid was present, in large amount in several date varieties.^14,15^ Caffeic, ferulic, 4-hydroxybenzoic and chlorogenic acids are some of the compounds detected in many date varieties, besides different flavonoids and derivatives like: rutin, catechin, quercetin, luteolin, vanillin and coumarin,^14,16^ and these results are in agreement with ours.

### 3.2. Scavenger effect of extracts against the DPPH radical

**Fig. 2:**
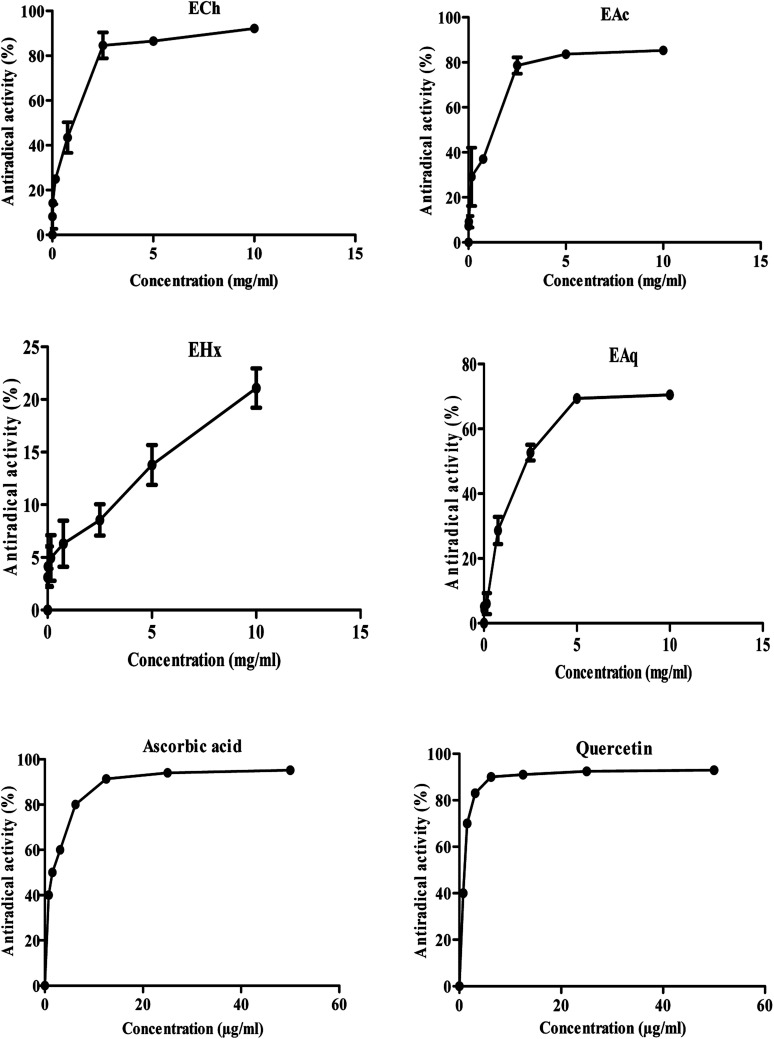
Maximum rate of anti-free radical activity of Tanteboucht extracts and standards.

Fig. 2 represents the profiles of the anti-free radical activity obtained for the various extracts of Tanteboucht besides that of ascorbic acid and quercetin standards. The IC_50_ and AE of each of extracts and standards were also determined (Table 2).

**Table 2:** Results of IC_50_ and antiradical efficiency (AE) of Tanteboucht extracts *versus* antioxidant standards

Extracts/standards	IC_50_ (mg mL^−1^)	AE
EAc	1.19 ± 0.02^a^^b^	0.83 ± 0.02^a^^b^
ECh	0.86 ± 0.08^a^^b^	1.16 ± 0.11^a^^b^
EAq	2.84 ± 0.90^a^^b^	0.37 ± 0.12^a^^b^
EHx	—	—
Quercetin	0.07 ± 0.01	14.48 ± 0.32
Ascorbic acid	0.25 ± 0.02	4.01 ± 2.09

a 1st letter: comparison (*p* <0.001) of all samples to quercetin standard.

b 2nd letter: comparison (*p* <0.001) of all samples to ascorbic acid standard.

These results revealed that all the extracts tested had an anti-free radical activity at the maximum concentration (10 mg mL^−1^); ECh exhibited the highest anti-free radical activity (92.22%), followed by EAc (85.25%) and then EAq (70.5%). The IC50 of DPPH of EHx could not be really quantified because at the maximum concentrations tested, only a fraction of 21% of DPPH was captured, indicating a low concentration of antioxidants in this extract. Moreover, ECh was found to be the most active extract with an IC_50_ of 0.86 mg mL^−1^ and an AE estimated at 1.16 followed by EAc with an IC_50_ of around 1.19 mg mL^−1^ and an AE of 0.83. The EAq gave the lowest anti-free radical activity with an IC_50_ of 2.84 mg mL^−1^ and an AE of around 0.37.

For comparative purposes, ascorbic acid and quercetin were used as antioxidant standards; they showed an interesting anti-free radical activity with an IC_50_ of the order of 0.25 and 0.07 mg mL^−1^, therefore an AE of the order of 4.01 and 14.48 respectively.

In comparison with ascorbic acid and quercetin all the extracts tested were found to be less active. Noting that, these standards are pure while the extracts are of crude composition.

Ghiaba *et al.*^17^ found IC_50_ equal to: 10.83, 13.20, 16.77 mg L^−1^ for methanolic extracts of the varieties Deglet Nour, Ghars, and Degla Baidha, these values are higher than those of the variety Tanteboucht; which indicates an antiradicalaire efficiency lower than that of Tanteboucht extracts. Noting that, these are not only different species but also the combined action of the different compounds with anti-free radical activity that they may contain.

Antioxidant molecules such as ascorbic acid, tocopherol, flavonoids and tannins have been shown to reduce and discolor DPPH, due to their ability to transfer the hydrogen atom and single electron.^18^ The polyphenols in the date extracts are probably responsible for the antioxidant activity of these extracts. Indeed the phytochemical investigation on Tanteboucht revealed its considerable contents of several flavonoids and phenolic acids besides tannins which may contribute to the pharmacological properties of this fruit. Substantial linear coefficients of determination (*R*^2^) of 0.71, 0.68 and 0.73, were obtained between the anti-free radical efficacy (EA) and the total identified phenolics, phenolic acids and tannins contents respectively. Furthermore, a very significant coefficient of determination of 0.96 existed between AE and flavonoid content. It is evident therefore, that the high activity of the ECh and EAc extracts is attributed in large part to phenolic acids, flavonoids and tannins detected in Tanteboucht extracts.

Noting that, Alam *et al.*^19^ conclude that the antioxidant activity depends on the polymerization degree as well as the hydroxylation of phenolic compounds, when studying this activity in several date varieties. Phenolic acids such gallic and chlorogenic acids play an important role in antioxidant reactions; these compounds can increase levels of superoxide dismutase, glutathione and catalase, which enhance considerably antioxidant defense mechanisms.^20,21^ On the other hand, Sato *et al.*^22^ reported that antioxidant activity of caffeic acid is stronger than that of its precursor chlorogenic acid.

In the same way, Shim *et al.*^23^ found that *p*-hydroxybenzoic acid had strong scavenging activity of DPPH radical. It was mentioned that vanillin had potent neuroprotective effect against oxidative brain damage.^24^ Coumarins are aromatic compounds, having good antioxidant capacity; which was reported in several studies.

Khalil and Mustafa^25^ found that isolated coumarins from Granny Smith apple seeds had considerable free radicals scavenging capacity. They were able to enhance antioxidant defence and thus improve tomato plant tolerance to salinity in the study of Antonijević *et al.* (2021).^26^ Indeed, they were found to be good inhibitors, with flavonoids, of peroxyl radicals.^27^

Rutin and quercetin are considered from the most potent antioxidant agents and several studies^28,29^ reported that these flavonoids may considerably reduce oxidative stress and lipid peroxidation in brain, kidney and liver by enhancing the expression of key genes involved in antioxidant processes like glutathione S transferase α (GSTα), paraoxonase-1(PON-1) and glutamate-cysteine ligase (GCL). This process will considerably decrease the plasma level of malondialdehyde (MDA) and Glutathione (GSH) considered as the principal markers of oxidative stress.^30^

Otherwise, quercetin and catechin have protective effect against collagen fragmentation, premature skin cancer and skin photo-aging processes^31,32^ by significantly reducing and neutralizing free radical and oxygen-mediated damage in cells and also in extracellular matrix.^33^ The pre-treatment with cyanidin chloride provided a neuroprotective effect againt ROS in a study on the nematode *Caenorhabditis elegans* (*C. elegans*).^34^ Otherwise, a strong correlation was obtained between antioxidant properties and cyanidin and tannins content of blackberry.^35^ Luteolin was found to ameliorate the activities of antioxidant enzymes such as superoxide dismutase, catalase, glutathione peroxidase, and glutathione reductase^36^ and thus showed a high activity in antioxidant process.

A research study highlighted antioxidant properties of this molecule against pro-oxidant effect at higher doses.^37^ These activities occurred by interacting with H_2_O_2_ and metal ions.

### 3.3. Antimicrobial activity

**Fig. 3:**
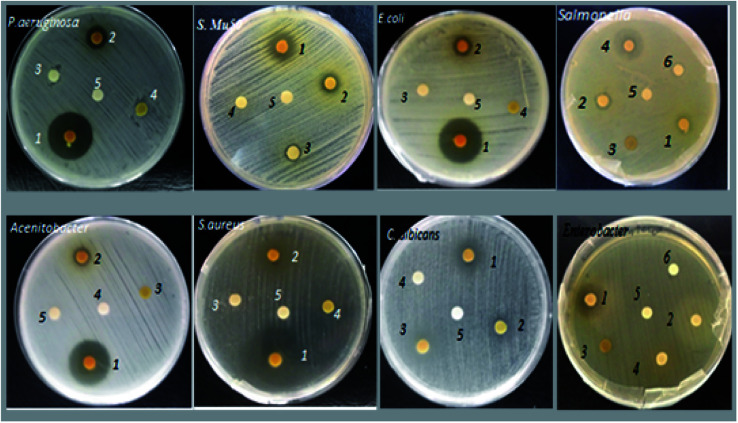
Major effects of Tanteboucht extracts on the sensitivity of the microbial studied strains; 1: EAc, 2: ECh, 3: EHx, 4: EAq, 5: DMSO, 6: Sterile distilled water.

Fig. 3 shows the majority of effects of the aqueous and organic extracts on the microbial studied strains. The results showed that under these experimental conditions, all the extracts had an inhibiting effect on at least one of the microbial strains tested. Indeed, all organic extracts of Tanteboucht exhibited an antimicrobial activity which depend on the bioactive molecules they contain, essentially the extract of ethyl acetate which seems to have the greatest activity against Gram (+) and Gram (−) bacteria; knowing that the most sensitive bacteria were *Staphylococcus aureus* and *P. aeruginosa* with a diameter of 22 mm followed by *E. coli* with a diameter of 20 mm, *Acinetobacter* 19.75 mm and finally *Enterobacter* with 10.55 mm (Table 3). The inhibition effect of EAc against *S. aureus*, *P. aeruginosa and E. coli* was considerable and comparable to some synthetic antibiotics used as standards (Table 3).

**Table 3:** Means diameters (mm) of inhibition zones of studied microbial strains by Tanteboucht date's extracts (200 mg mL^−1^) and standards

Extract/strain	EAc	EHx	ECh	EAq	Standards
*Enterobacter Cancerogenus*	10.55 ± 0.20	—	6.60 ± 0.35	6.55 ± 0.41	—
*MRSA*	7.75 ± 0.37	—	6.70 ± 0.43	—	—
*Staphylococcus Mu50*	10.90 ± 0.33	8.00 ± 0.05	9.50 ± 0.11	—	—
*Salmonella typhimurium*	7.30 ± 0.21	—	9.20 ± 0.26	16.75 ± 0.30	19 (cefoxitine)
					22 (imipenem)
*Acinetobacter Sp*	19.75 ± 0.45	—	8.00 ± 0.15	—	—
*E. coli*	20.00 ± 0.32	—	8.50 ± 0.45	—	20 (amoxicilline)
					25(Gentamycine)
*Staphylococcus aureus*	22.00 ± 0.38	8.00 ± 0.5	9.00 ± 0.16	—	19 (oxacilline)
					19 (vancomycine)
*Bacillus polyma*	10.60 ± 0.15	—	—	—	—
*P. aeruginosa*	22.00 ± 0.37	—	9.00 ± 0.35	—	10 (tetracycline)
					20 (Gentamycine)
*C. albicans*	10.00 ± 0.18	8.50 ± 0.13	8.50 ± 0.18	—	—

ECh gave a lower antibacterial activity compared to EAc and whose most sensitive strains were *Staphylococcus Mu50* and *S. typhimurium* with inhibition diameters of 9.5 and 9.2 mm respectively; followed by *P. aeruginosa* and *Staphylococcus aureus* which gave 9.00 mm and finally *E. coli* and *Candida albicans* with 8.5 mm of inhibition diameter. However, it did not give any activity against *Bacillus polyma*. Daas amiour *et al.*^12^ found that alcoholic extracts from three varieties of dates had minimal inhibitory effect against the *Escherichia coli* strain which agree with this result.

EHx has been shown to be active only against *Candida albicans* (8.5 mm), *Staphylococcus aureus* and *Staphylococcus Mu50* both with 8 mm as the diameter of inhibition zone. This activity may be due to the richness of this extract in lipid compounds which can hinder microbial growth. Indeed according to Branen *et al.*^38^ long chain fatty acids have interesting antibacterial effects.

The aqueous extract gave a good zone of inhibition against Gram (−) strains mainly *S. typhimurium* (16.75 mm), however, it gave minimal effect with Enterobacter. This activity can be explained by the richness of this extract in tannins which are capable of combining with microbial enzymes and of chelating certain metals such as iron,^39^ thus stopping microbial growth.

The fungal strain *C. albicans* appears to be sensitive to all organic extracts with diameters of 10 mm, 8.5 mm, 8.5 mm for EAc, ECh and EHx respectively, it may be due to the presence of phenol acids and flavonoids in these extracts. Indeed, Branen *et al.*^38^ confirmed that phenolics have both antibacterial and antifungal activity. In contrast, no inhibition of *Candida albicans* was observed with the aqueous extract.

To ensure that the activity is intrinsic to the extracts, the activities of the solvents used for the extraction and that used for the dissolution (DMSO) of the organic extracts were tested and gave negative results, one concentration initially. To determine the minimum inhibitory concentration (MIC) of EAc extract which had the most potent antimicrobial effect with maximum diameter of inhibitory zone of 22 mm (Table 3), dilutions (50%, 25%, 12.5%, 6.25%, 3.12%) were tested and the results are mentioned in Table 4.

**Table 4:** Diameters of inhibition zones (mm) of *Staphylococcus aureus*, *Escherichia coli* and *C.albicans* obtained at different concentrations of the ethyl acetate extract (EAc)

Strain/dilution	50	25	12.5	6.25	3.12
*S. aureus*	18.2 ± 0.1	12.9 ± 0.16	10.75 ± 0.08	10.00 ± 0.01	6.5 ± 0.01
*E. coli*	17.78 ± 0.47	14.22 ± 0.02	11.93 ± 0.10	10.53 ± 0.20	6.75 ± 0.02
*C. albicans*	15.55 ± 0.04	11.02 ± 0.02	06.73 ± 0.09	—	—

According to the results obtained by HPLC, the ECh and EAc extracts from Tanteboucht contain several flavonoids and phenolic acids, which have significant antibacterial activity.^40^ Indeed, this author signalled that these molecules could exert antibacterial effects since they are powerful *in vitro* as inhibitors of DNA gyrase. He added that they have antimicrobial activity attributed to their phenolic function, this activity is supposed to increase with number of hydroxyl, methoxyl or glucosyl substituents and according to Mansouri *et al.*^11^ these types of flavonoids are present in dates.

The mechanism of the antimicrobial effects of polyphenols is undoubtedly very complex, among the hypotheses put forward, we can cite: Inhibition of extracellular microbial enzymes, the sequestration of the substrate necessary for microbial growth, the chelation of metals such as iron and the inhibition of microbial metabolism.^40^

The correlation results between the content of phenolic compounds and the antimicrobial effect of the extracts showed that the inhibitory effect exerted on *S. Mu50*, *C. albicans* and *Enterobacter cancerogenus* by the hexane, chloroform and ethyl acetate extracts seems to be due to flavonoids, given that there is a strong correlation between the content of flavonoids and the inhibitory effect whose correlation coefficients are (*R*^2^ = 0.98), (*R*^2^ = 0.89), (*R*^2^ = 0.85), respectively. Tannic acid was found as inhibitor of both Gram-positive and Gram-negative bacteria, such as: *Staphylococcus aureus*, *Escherichia coli*, *Streptococcus pyogenes*, *Enterococcus faecalis*, *Pseudomonas aeruginosa*, *Yersinia enterocolitica, Listeria innocua*.^41^

Coumarin was found active on *Escherichia coli* 81nr.149 SKN541, *Enterobacter aerogenes* CIP 104 725, *Salmonella typhimurium* SKN533 and *Salmonella infantis* SKN 557 in the study carried out by Nitiema *et al.*,^42^ where minimum inhibitory concentration of this molecule was from 0.625 to 5.0 mg mL; however, the minimum bactericidal concentration was ≤5 mg mL^−1^. Additionally, Rutin from *Peumus boldus* extract had highest antibacterial activity, especially against *E. coli* and *Bacillus cereus*.^43^

On the other hand, it was signalled in the study of Gutiérrez-Venegas *et al.*^44^ that luteolin, quercetin and rutin were actives against *Escherichia coli, staphylococcus aureus* and *Candida albicans* while catechin inhibited only *staphylococcus aureus.* It was found also in the study of Tyagi *et al.*^45^ that gallic, caffeic and tannic acids besides catechin and quercetin have considerable inhibition of strains of *Escherichia coli* and *Pseudomonas aeruginosa.*

Furthermore, it was reported by Paz *et al.*^46^ that, identified phenolics in extracts of *Hamelia* patens were able to inhibit *Escherichia coli*, *Staphylococcus aureus* and *Salmonella typhi* growth. This is because, these phenolic compounds contain hydroxycinnamic acid, quercetin, procyanidin B2, catechin besides epicatechin; therefore, they concluded that the last compound was more implicated in this activity.

### 3.4. Membrane stabilizing effect

**Fig. 4:**
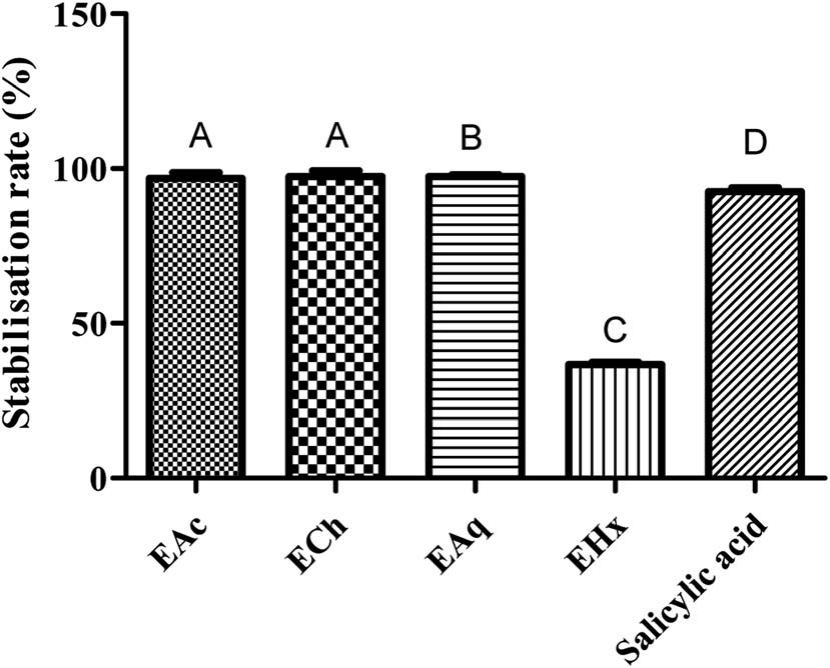
Rate of anti-inflammatory activities of Tanteboucht extracts *vs.* standard (Salicylic acid). Results are the average of three measurements; the bars with different letters indicate significantly different activities (*p* <0.001).

The results showed that all Tanteboucht extracts exhibited membrane stabilizing activity using concentration of 2 mg mL^−1^, essentially the ECh which had the greatest activity with a degree of 99.25 ± 0.08% of membrane stabilization, followed by the EAc with a percentage of 98.85 ± 0.12%, then the EAq with 98 ± 0.13% and finally the hexane extract giving 37 ± 0.19% as a stabilization rate (Fig. 4). The activity of the first three extracts is very important and significantly higher than that obtained with the positive control which gave an activity of 93%.

The significant membrane stabilizing activity of extracts from the Tanteboucht date can be attributed to the identified flavonoids, in particular flavonols and flavanols, as well as tannins; indeed, examination of these results revealed a significant linear coefficient of determination (*R*^2^ = 0.64) between membrane stabilizing effect and flavonoid content and a very significant coefficient of determination (*R*^2^ = 0.99) between this activity and tannin content. According to Lavanya *et al.*^47^ all these compounds are endowed with anti-inflammatory power.

The obtained results allow us to conclude that Tanteboucht phenolics are involved in the membrane stabilization capacity of its extracts which decreases the possibility of installation of inflammation as it was indicated in several studies that inhibition of erythrocyte membrane damage is considered to be an index of anti-inflammatory activity.^48,49^

In the study of Yin *et al.*,^50^ Procyanidin B2 from grape seed showed an anti-inflammatory activity and thus provided protective effect against the damage of the diabetic pancreas. Lesjak and his collaborators^51^ carried out a study on antioxidant and anti-inflammatory activities of quercetin and its derivatives and concluded that, these compounds may act as effective anti-inflammatory and antioxidant agents. Soyocak *et al.*^52^ signalled that tannic acid may contribute to the treatment of inflammation by decreasing myeloperoxidase activity in rats against formalin-induced paw oedema. Hence, the *p*-hydroxybenzoic acid found abundantly in Tanteboucht extracts has potent biological activities, as previously confirmed in several studies. Indeed, this molecule reduced oxidative stress induced by hydrogen peroxide and contributed in inhibition of neurodegeneration process, which confirm its anti-inflammatory effect in the study conducted by Winter *et al.*^53^ On the other hand, Maleki *et al.*^54^ mentioned that flavonoids have beneficial properties *in vitro* in inflammatory diseases and they can inhibit enzymes or transcription factors involved in inflammation process.

## 4. Conclusion

Antioxidant, anti-inflammatory and anti-microbial studies were investigated in this work, and results showed important effects of Tanteboucht extracts, especially ethyl acetate and chloroformic extracts. Indeed, EAc and ECh were more potent in inhibition of microbial strains and EAc showed comparable efficiency to antibiotics. Furthermore, considerable scavenging activity was obtained with these extracts but it was less than the standards one. On the other hand, EAC, ECh and EAq exhibited very important rates of anti-inflammatory activity which was significantly higher than that of the positive control. The obtained considerable activities were significantly correlated with flavonoids and tannins contents in the extracts. Tanteboucht date must be valued, given its pharmacological properties and further investigations are needed to clearly understand the full potential of this fruit.

## Author contributions

Conceptualization, Saliha Dassamiour; data curation, Saliha Dassamiour, Selsabil Meguellati and Hdouda Lamraoui; Formal Analysis, Saliha Dassamiour and Mohamed Sabri Bensaad; Funding acquisition, Rokayya Sami; Investigation, Saliha Dassamiour and Selsabil Meguellati and Hdouda Lamraoui; Methodology, Saliha Dassamiour and Mohamed Sabri Bensaad; Project administration Saliha Dassamiour and Rokayya Sami; Resources, Saliha Dassamiour and Rokayya Sami; Software, Saliha Dassamiour; Supervision, Saliha Dassamiour and Rokayya Sami; Validation, Saliha Dassamiour, Mohamed Sabri Bensaad and Rokayya Sami; Visualization, Saliha Dassamiour, Mohamed Sabri Bensaad, Rokayya Sami, Garsa Alshehry, Eman Hillal Althubaiti and Areej Suliman Al-Meshal; Writing – original draft, Saliha Dassamiour, Selsabil Meguellati and Hdouda Lamraoui; Writing – review & editing, Saliha Dassamiour, Mohamed Sabri Bensaad, Rokayya Sami, Garsa Alshehry, Eman Hillal Althubaiti and Areej Suliman Al-Meshal.

## Conflicts of interest

The authors declare no conflicts of interest.

## Ethical statement

The authors of the publication declare that the patients/participants have been informed that their blood will be used for testing, provided their written informed consent to participate in this study and agreed to use the research results to prepare a scientific publication.

## Supplementary Material
